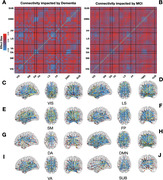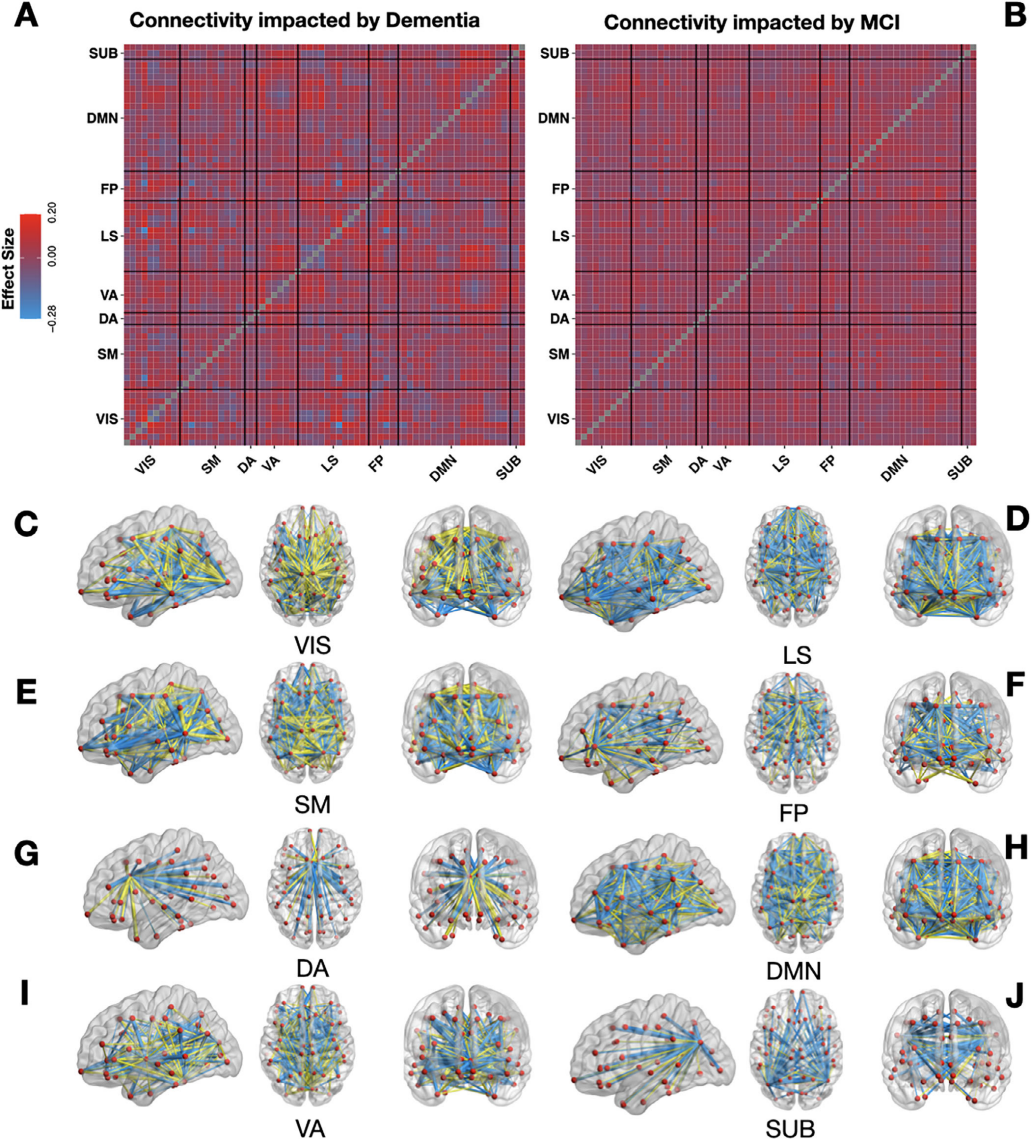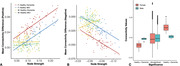# Sex‐specific topological structure associated with dementia identified via latent space network analysis

**DOI:** 10.1002/alz.090404

**Published:** 2025-01-09

**Authors:** Selena Wang, Yiting Wang, Fredericks Xu, Li Shen, Yize Zhao

**Affiliations:** ^1^ Yale, New Haven, CT USA; ^2^ Univeristy of Virginia, Charlottesville, VA USA; ^3^ Univeristy of Pennsylvania, Philadelphia, PA USA; ^4^ University of Pennsylvania, Philadelphia, PA USA; ^5^ Yale University, New Haven, CT USA

## Abstract

**Background:**

Statistical network analysis has transformed neuroimaging research in recent years by enabling flexible and intuitive integration of multiple data types and preserving the topological brain connectivity structure while uncovering mechanism of degenerative aging. In this study, we apply a novel latent space joint network model to perform a case‐control comparison using the functional connectivity data together with region‐specific cortical volume, cortical thickness, surface area and PET information. By preserving complex network structures during imaging biomarker detection, we find sex‐specific topological structures associated with dementia.

**Method:**

We used resting state functional neuroimaging data from the ADNI study collected from 406 subjects, 208 males and 198 females. Of these, 198 subjects are healthy; 158 subjects show mild cognitive impairment; and 55 subjects show symptoms of dementia. To capture the group‐level functional connectivity information, the proposed method extends the latent space model to accommodate multiplex brain network structure and identify significant imaging biomarkers by comparing group‐level connectivity between healthy, MCI and dementia patients.

**Result:**

For female subjects, areas of connectivity edges that are impacted by dementia and MCI tend to following the organizational topological structure of the brain (Figure 1). In contrast, areas of connectivity edges that are impacted by dementia and MCI for the male subjects do not follow such structures (Figure 2). Furthermore, we found that, among the female subjects, the difference between healthy and patient population tends to be larger for central actors of the network, brain regions of high node strength (Figure 3A and B). In Figure 3C, we see that, for females, there seem to be differences in brain connectivity among different types of imaging biomarkers, but not for males. For females, strong connectivity edges tend to indicate the development of dementia; whereas, for males, there is no such trend.

**Conclusion:**

The proposed latent space joint network model is designed to capture brain topological structure while performing statistical inference. This initial AD functional connectivity study demonstrates the promise of uncovering the topological structure associated with degenerative aging and foster future investigations of the underlying mechanisms between network structures and data modalities and provide a new lens to disease pathology.